# Apoptotic investigation of brain tissue cells in dogs naturally infected by canine distemper virus

**DOI:** 10.1186/s12985-021-01635-8

**Published:** 2021-08-12

**Authors:** Yaoqian Pan, Shuai Wang, Peng Li, Feng Yue, Yanfang Zhang, Bo Pan, Xingyou Liu

**Affiliations:** 1 School of Life Science and Basic Medicine, Xinxiang University, Xinxiang, 453003 Henan China; 2 School of Basic Medical Sciences, Xinxiang Medical University, Xinxiang, China; 3 Division of Basic Biomedical Sciences, Sanford School of Medicine of the University of South Dakota, Vermillion, SD 57069 USA

**Keywords:** Apoptosis, Canine distemper virus, Astrocyte, Oligodendrocyte, Demyelination

## Abstract

**Background:**

Canine distemper caused by canine distemper virus that belongs to the *Morbillivirus* genus of the Paramyxoviridae family is still a global epidemic significant infectious disease, especially in pet dogs in China and serious harm to the development of the dog industry. It has been known that apoptosis caused by the canine distemper virus can show in culture cells, lymphoid tissues, and the cerebellum. However, its occurrence in brain tissue cells remains unclear. To investigate the relationship among canine distemper infecting brain tissues, apoptosis in brain tissue cells, and demyelinating pathogenesis was investigated.

**Methods:**

16 naturally infected dogs that exhibited clinical signs of CD and tested positive for the anti-CDV monoclonal antibody and six healthy dogs that served as the control, were used in the research. Brain specimens were divided into the cerebrum, brain stem, and cerebellum embedded in paraffin and made the sections respectively. Approximately 5 µm-thick sections were stained by hematoxylin–eosin, methyl green pyronin, terminal deoxynucleotidyl transferase-mediated dUTP nick-end labeling technique, and immunohistochemistry. CDV nucleocapsid protein was detected by immune streptavidin-biotinylated peroxidase complex.

**Results:**

Alterations in the brain tissues of CDV-infected dogs involved both various cells and nerve fibers. CDV had varying degrees of cytotropism to all brain tissue cells; apoptosis also occurred in all brain cells, especially in the endothelia of cerebral vessels, astrocytes, oligodendrocytes, and ependymal cells, the more serious infection, the more obvious apoptosis. Serious infections also involved the pyramidal and Purkinje cells. The nervous fibers exhibited demyelinating lesions (showed small multifocal vacuole), and some axonal neuron atrophy gradually disappeared (formed large vacuole).

**Conclusions:**

Apoptosis in brain tissue cells was mainly related to the propagation path and cytotropism of CDV. The apoptosis of astrocytes, oligodendrocytes, and some neurons may play a significant role in the demyelinating pathogenesis in dogs with acute canine distemper. A lot of diverse nervous signs shown in the clinic may be related to different neuron apoptosis.

## Introduction

Canine distemper (CD) is still a global epidemic significant infectious disease caused by canine distemper virus (CDV) that belongs to the *Morbillivirus* genus of the Paramyxoviridae family [1–3]. The natural host spectrum of CDV comprises at least six orders and over 20 families of mammals [4, 5], in particular all families of the order Carnivora [6, 7], including dogs that are susceptible to infection of CDV [8]. According to recent reports [9–12], the risk of re-emergence of canine distemper presents in the world, and some new strains of canine distemper virus have been isolated in Asia. It seems to tell us must pay attention to the prevention and control of CD at present.

The pathogenesis of CD mainly involves the central nervous system and immunosuppression. The damage in the central nervous system (CNS) is an important characteristic of CDV-infected dogs. Demyelinating encephalopathy is the most common histopathological lesion of CD in the early or acute stage of the disease [13–15]. Pathologically, CNS alterations in CD frequently involve both white matters that shows multifocal or diffusive demyelination in the nerve fiber bundle and grey matter that mainly displays diverse degeneration of the neurons. Glial cell infection precedes demyelination and is correlated to strong viral replication in the glial cells of white matter [16]. Nervous signs are the most common clinical manifestations in dogs with CD, including apathy, stupor, behavior disorder, seizures, ataxia, tetraparesis, tetraplegia, paraplegia, hyperesthesia, myoclonus, and incontinence [17–20]. Although dogs with CD often have diverse neurological manifestations, they are difficult to diagnose based only on neurological signs in clinics.

According to the previous study, CDV has been shown to induce apoptosis in vivo, such as in the cerebellum [21] and lymphoid tissue of naturally infected dogs [22, 23]. CDV also causes apoptosis in vitro, such as in Vero [24, 25] and HeLa cells [26]. However, apoptosis in the brain of dogs naturally infected with CDV remains unclear. This study aimed to determine whether CDV induced apoptosis in naturally infected dogs' brains and elucidate the apoptotic relationship between CDV infection and demyelinating pathogenesis in the brain.

## Methods

### Animals

The 22 dogs used in this research were divided into two groups. Group 1 comprised 16 dogs with neurological signs (Nos. 1–16, experimental group) and suffering from acute spontaneous CD diagnosed by anti-CDV monoclonal antibody in the clinic. Group 2 consisted of six healthy Beagle dogs (three males and three females, age 6 months, Nos. 17–22, control group) that served as control animals from a study on traditional Chinese herb medicine. These dogs were vaccinated and dewormed according to standard protocols.

### Sample collections and histology

Brains were immediately collected from animals at necropsy and fixed by immersing in 10% neutral-buffered formalin for 72 h. The fixed brain was split into left and right hemispheres along the longitudinal fissure to observe the brain lesions in detail and then divided into the cerebrum, brain stem, and cerebellum and sliced into 0.3 cm thickness. All tissues were dehydrated through gradient alcohols, embedded in paraffin, and sectioned at 5 μm thicknesses by Leica microtome. The sections were stained by hematoxylin–eosin (HE). Control animals (Nos. 17–22) were mercy killing, and their brains were subjected to the same histopathological procedures.

### In situ detection of genome fragmentation

The terminal deoxynucleotidyl transferase-mediated dUTP nick-end labeling (TUNEL) technique was performed for in situ detection of DNA fragmentation using a commercial ApopTag Plus in situ apoptosis detection kit (DAKO EPOS). The reaction was carried out according to the manufacturer's descriptions. In brief, the sections were incubated with proteinase K at room temperature (RT) for 15 min, and endogenous peroxidase was quenched with 3% H_2_O_2_ in methanol at RT for 5 min. Terminal deoxynucleotidyl transferase and deoxynucleotides were applied, then the sections were placed in a humid atmosphere at 37 °C for 30 min. The reaction was stopped by a blocking buffer. The sections were treated with peroxidase, streptavidin, conjugate plus diaminobenzidine (DAB), and lightly counterstained with hematine.

### Methyl green–pyronin (MGP) stain

MGP stain was used to elucidate the DNA and RNA change in the nucleolus and cytoplasm when apoptosis occurred. The sections from the brain tissues were deparaffinized, hydrated with distilled water, covered with MGP solution (Bi Yun-Tian, China) for 4 min, then blocked and air-dried at RT for 5 min, differentiated with distilled water, dehydrated, and mounted.

### Detection of CDV by immunohistochemistry

The streptavidin-biotinylated peroxidase complex (sABC) method was used by a monoclonal antibody against CDV nucleocapsid protein (NP) to detect the CDV antigens to confirm the CDV infection. Sections were autoclaved in citrate buffer at 121 °C for 5 min for anti-CDV immunostaining. The endogenous peroxidase activity was quenched with 3% (w/v) H_2_O_2_ diluted in methanol at RT for 10 min. The sections were then incubated with primary antibody (mouse anti-CDV monoclonal antibody 1:400; Chemicon International, Inc.) at 4 °C for 24 h and secondary antibodies (biotinylated goat anti-mouse and rabbit IgG; Chemicon International, Inc.) at RT for 10 min. Finally, the sections were visualized with DAB and lightly counterstained with hematoxylin.

### Anti-glial fibrillary acidic protein (GFAP) assay

The anti-GFAP assay was performed to investigate the metabolism of astrocytes, in which GFAP is expressed only by astrocytes and is considered a specific marker for astrocytes. In brief, the rehydrated sections were dipped in 3% hydrogen peroxide in methanol solution at RT for 15 min, blocked with normal goat serum diluted (1:20) in PBS at RT for 20 min, incubated with primary antibody (mouse monoclonal anti-GFAP; DAKO EPOS) at RT for 80 min, incubated with secondary antibody (goat anti-mouse IgG peroxidase conjugate) at RT for 40 min, visualized by DAB and lightly counterstained with hematoxylin.

### Anti-galactocerebroside (GalC) assay

Anti-GalC assay (GalC detection kit; DAKO EPOS) was used to examine oligodendrocytes' type and metabolic state. In brief, the deparaffinized-rehydrated sections were dipped in 3% hydrogen peroxide in methanol solution at RT for 10 min, autoclaved in citrate buffer at 121 °C for 5 min, incubated with primary antibodies at RT for 60 min, added with the secondary antibody at RT for 30 min and visualized with DAB.

## Results

During examination in the clinic, all 16 dogs with natural CD displayed diverse neurological manifestations ranging in severity. Some dogs showed mild signs, whereas others developed serious features similar to those [15, 16, 20] reported for CD. The most common manifestations were apathy (16/16), anorexia (16/16), myoclonus (16/16), seizures(12/16), ataxia (10/16), tetraparesis (8/16), nystagmus (7/16), pedal movements (5/16), and opisthotonos (5/16) (Table 1). The control animals showed a negative reaction to an antibody against CDV. None of the control animals displayed any neurological signs.

**Table 1:** General nature condition and neurological signs of 16 dogs with acute CD

Cases no.	Age (months)	Sex	Breed	Anti-CDV antibody	Main neurological signs in clinic
1	5	M	Mongrel	+	Spastic tetraparesis, cervical rigidity, myoclonus, apathy
2	7	M	Labrador	+	Myoclonus in bilateral thoracic limb, tetraparesis, anorexia
3	4	F	Beagle	+	Spastic tetraplegia, myoclonus in pelvic limb, apathy
4	6	M	Poodle	+	Myoclonus in truncal and limb, nystagmus, apathy, anorexia
5	4	F	Mongrel	+	Tremor, ataxia, myoclonus in truncal and limb, anorexia
6	6	F	Mongrel	+	Disorientation, myoclonus in pelvic limb, nystagmus
7	5	M	Shiba	+	Spastic tetraparesis, ataxia, myoclonus in pelvic limb
8	5	F	Shin Tzu	+	Spastic tetraplegia, myoclonus in thoracic limbs, apathy
9	3	F	Labrador	+	Apathy, pedal movement, disorientation, dementia
10	4	M	Shin Tzu	+	Apathy, pedal movement, disorientation, dementia
11	7	M	Mongrel	+	Spastic tetraplegia, myoclonus in pelvic limbs, seizures
12	4	M	Pekingese	+	Tremor, spastic paraparesis, ataxia, opisthotonus
13	5	F	Pekingese	+	Tremor, ataxia, myoclonus in pelvic limbs, pedal movement
14	7	F	Poodle	+	Myoclonus in cervical and thoracic limbs
15	6	M	Shin Tzu	+	Myoclonus in truncal and limb ataxia, apathy
16	5	M	Mongrel	+	Apathy, anorexia, seizures, nystagmus, opisthotonus

M, male; F, female; + , positive

Alterations in the brain tissues of CDV-infected dogs involved both various cells and nerve fibers. The changes in all kinds of brain tissue cells were mainly CDV infection, degeneration, and apoptosis. The nerve fibers mainly exhibited demyelinating lesions (showed small multifocal vacuole), and some neuron axonal atrophied and disappeared gradually (formed large vacuole). CDV had varying degrees of cytotropism in all brain tissue cells, and apoptosis also occurred in all kinds of cells in the brain. The more serious CDV infected, the more apoptotic cells occurred.

**Fig. 1:**
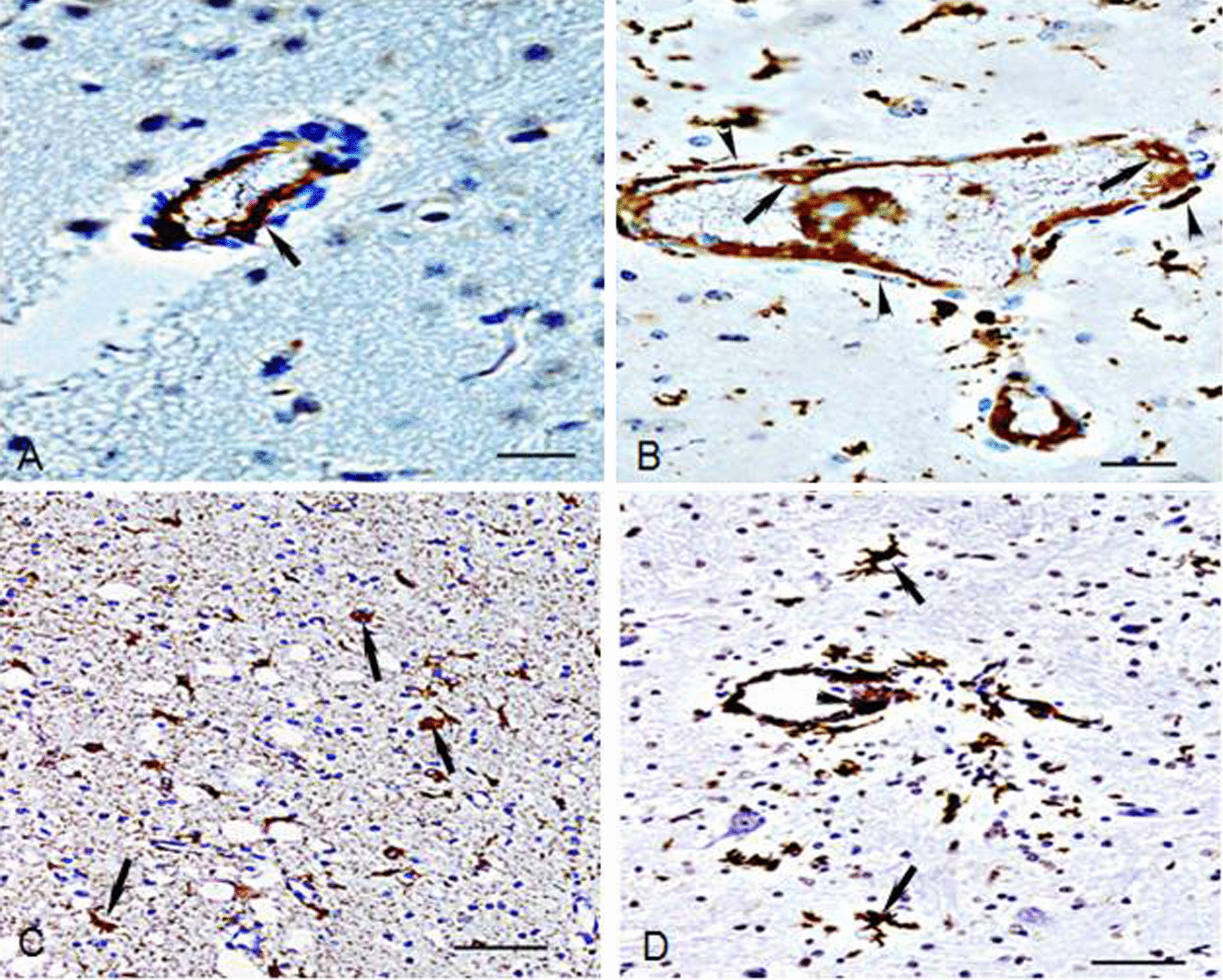
Severe CDV-infected endothelia and astrocytes **A** CDV antigen intense positive reaction in the endothelium (up arrow) of the blood vessel. sABC staining, Bar = 20 μm. **B** CDV antigen intense positive reaction of endothelia (up arrow) and astrocytic food processes (up arrow) surrounding the blood vessels and astrocytes. sABC staining, Bar = 20 μm. **C** Astrocytes located in mild and moderate demyelinating areas are intense positive reactions (up arrow) against GFAP. Anti-GFAP staining, Bar = 50 μm. **D** Apoptosis of endothelia (up arrow) and star-patterned astrocytes (up arrow) are detected in primary demyelinating areas. TUNEL staining, Bar = 50 μm

The severe CDV-infected cells in brain tissue were cerebral blood vessel endothelial, astrocytes, and ependymal cells. The intense positive reactive endothelial, showing brown cells or dark brown cells (Fig. 1A), were primarily examined in small vessels of brain tissues when the sections were stained by anti-CDV immunostaining. Following infectious exacerbation, the endothelium and astrocytic foot processes that appeared like line symbols and encircled in blood vessels showed intense positive reaction by anti-CDV immunostaining, suggesting that CDV had infected these cells. Astrocytes were also stained as intense positive cells and became darker brown star-like patterns (Fig. 1B) by anti-CDV immunostaining, indicating that CDV had infected astrocytes and started to replication in the cytoplasm of astrocytes. Anti-GFAP staining exhibited that the astrocytes in the demyelinating areas were in metabolic disorder and had several anti-GFAP strong positive substances (weak positive reaction in control dogs) in their cytoplasm (Fig. 1C). Some of the endothelia of small vessels and astrocytes around the blood vessels had a strong positive reaction (Fig. 1D) by TUNEL staining, showing dark brown cells and demonstrating their apoptosis.

MGP staining revealed that some nuclei of endothelia and astrocytes around the blood vessels showed pyknosis and appeared dark red, indicating they were in an apoptotic status because RNA of the nucleolus in these cells become to condensation.

**Fig. 2:**
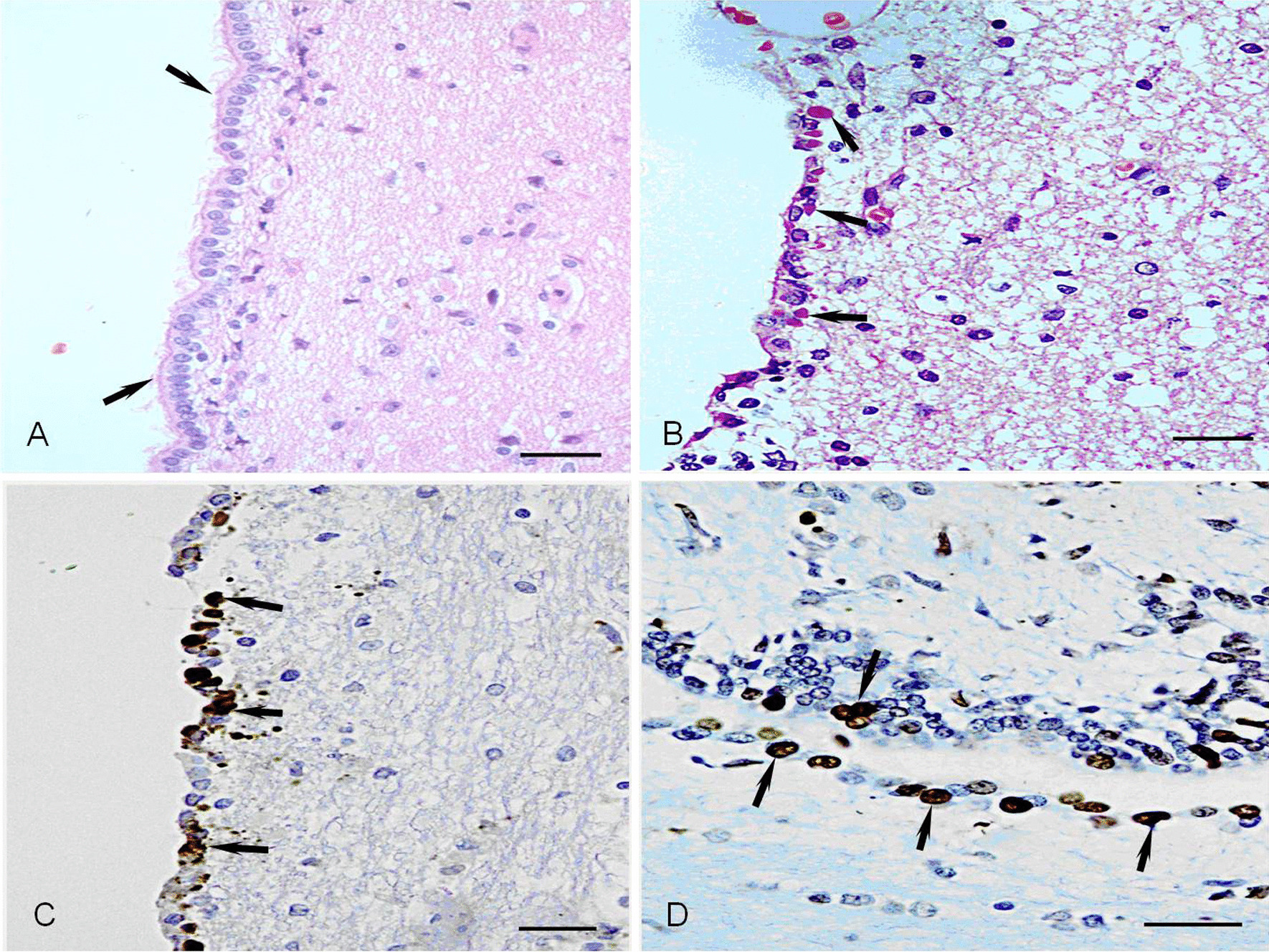
Severe CDV-infected ependymal cells. **A** Normal ependymal cells appear like columnar cells (up arrow) with microvilli on their free surface. HE staining, Bar = 20 μm. **B** There are some eosinophilic inclusions (up arrow) in the cytoplasm and nucleus of ependymal cells. HE staining, Bar = 20 μm. **C** CDV antigen intense positive reaction (up arrow) in ependymal cells with inclusions. sABC staining, Bar = 20 μm. **D** Many apoptotic cells (up arrow) are found among ependymal cells. TUNEL staining, Bar = 20 μm

The ependymal cells of the control animals appeared like columnar cells with microvilli on their free surface (Fig. 2A) and had a negative reaction with anti-CDV and TUNEL staining. However, in CDV-infected dogs, the ependymal cells became cubic or flat cells, in which microvilli in cellular membrane surface disappeared, and several eosinophilic inclusions were observed in their cytoplasm and nuclei (Fig. 2B). Anti-CDV positive reaction matter was observed in the eosinophilic inclusions of ependymal cells (Fig. 2C), proving that CDV had infected ependymal cells because the eosinophilic inclusions were produced by CDV replication and metabolism. When the cells were seriously infected, numerous ependymal cells died via apoptosis, in which the nucleus was stained dark brown (Fig. 2D) by TUNEL staining.

**Fig. 3:**
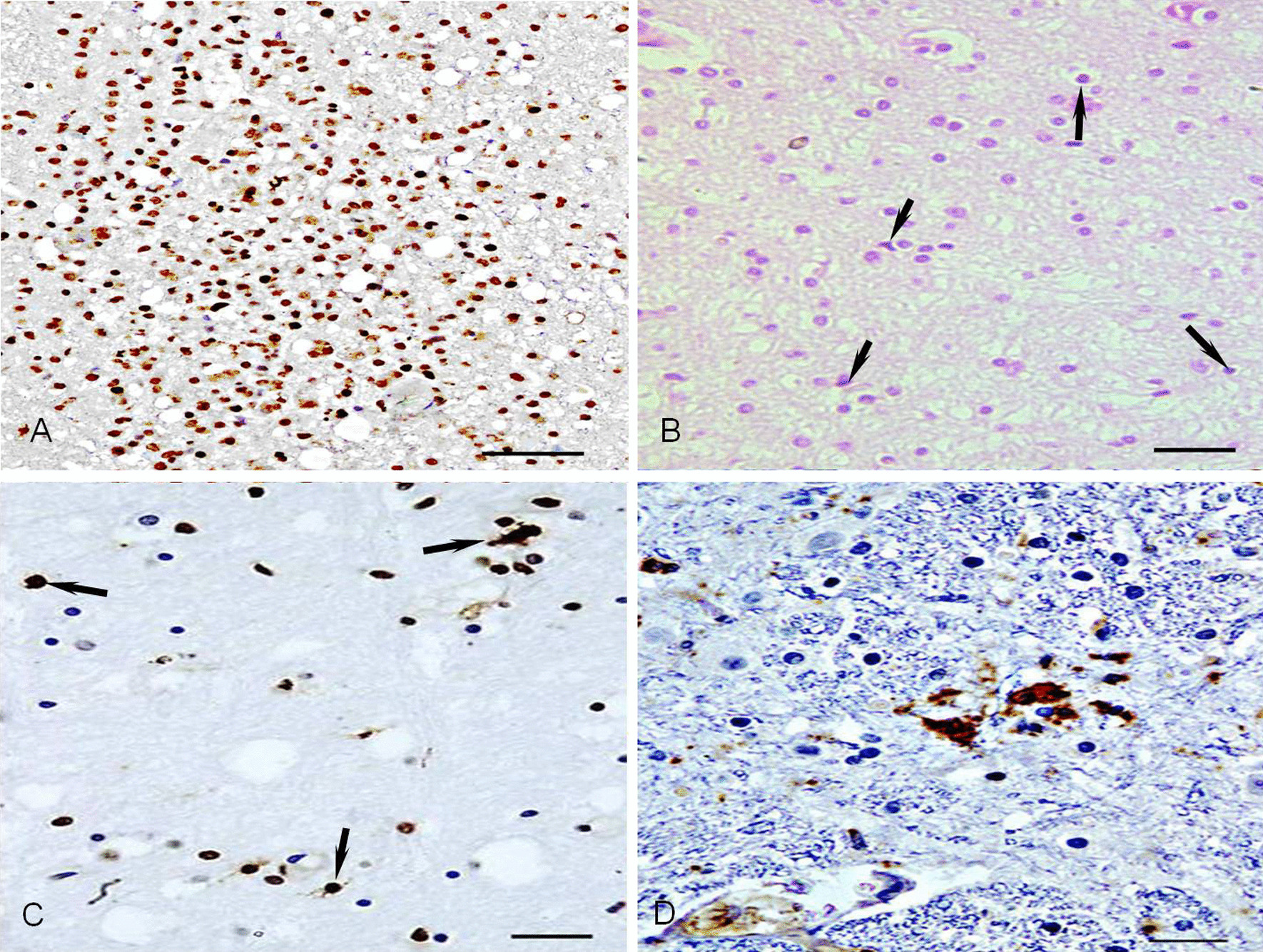
Moderate CDV-infected oligodendrocytes and nerve nucleus cells. **A** Oligodendrocytes show an anti-GalC strong positive reaction in demyelinating areas. Anti-GalC staining, Bar = 50 μm. **B** Some intense positive oligodendrocytes (up arrow) were observed in initial demyelinating areas. MGP staining, Bar = 50 μm. **C** A lot of apoptotic multipolar mature oligodendrocytes (up arrow) present in demyelinating areas. TUNEL staining, Bar = 50 μm. **D** Strong positive neurons for anti-CDV antigens in the nerve nucleus cells situated in white matter. sABC staining, Bar = 50 μm

The secondary CDV-infected cells in brain tissue were oligodendrocytes, microglia, and neurons mainly located in the nerve nucleus of white matter in the brain stem. The oligodendrocytes of the brain stem in the control dogs were arranged like a string of beads along the nerve fibers and exhibited a negative reaction for anti-CDV and TUNEL staining and a weak positive reaction for GalC staining. By contrast, in CDV-infected dogs, some oligodendrocytes in demyelinating areas were in a dispersive state and had a strong positive reaction for anti-GalC staining, which might indicate that bipolar immature oligodendrocytes increased (Fig. 3A). However, multipolar mature oligodendrocytes decreased in some initial demyelinating areas via apoptosis, showing an intensely positive reaction by MGP (Fig. 3B) and TUNEL staining (Fig. 3C). Following the apoptosis of oligodendrocytes, local and diffuse demyelination was observed in the white matter of the brain stem and cerebrum, showing lots of small vacuoles and bigger vacuoles in demyelinating areas stained HE. A few microglia were present in demyelinating areas and appeared among oligodendrocytes, some of which showed a strong positive reaction for anti-CDV and TUNEL staining. Some of the neurons in the nerve nucleus of white matter were often infected by CDV, in which shrunken neurons with condensed nuclei exhibited a strong positive reaction for anti-CDV (Fig. 3D) and TUNEL staining, indicating that neurons severely infected by CDV were in apoptosis.

**Fig. 4:**
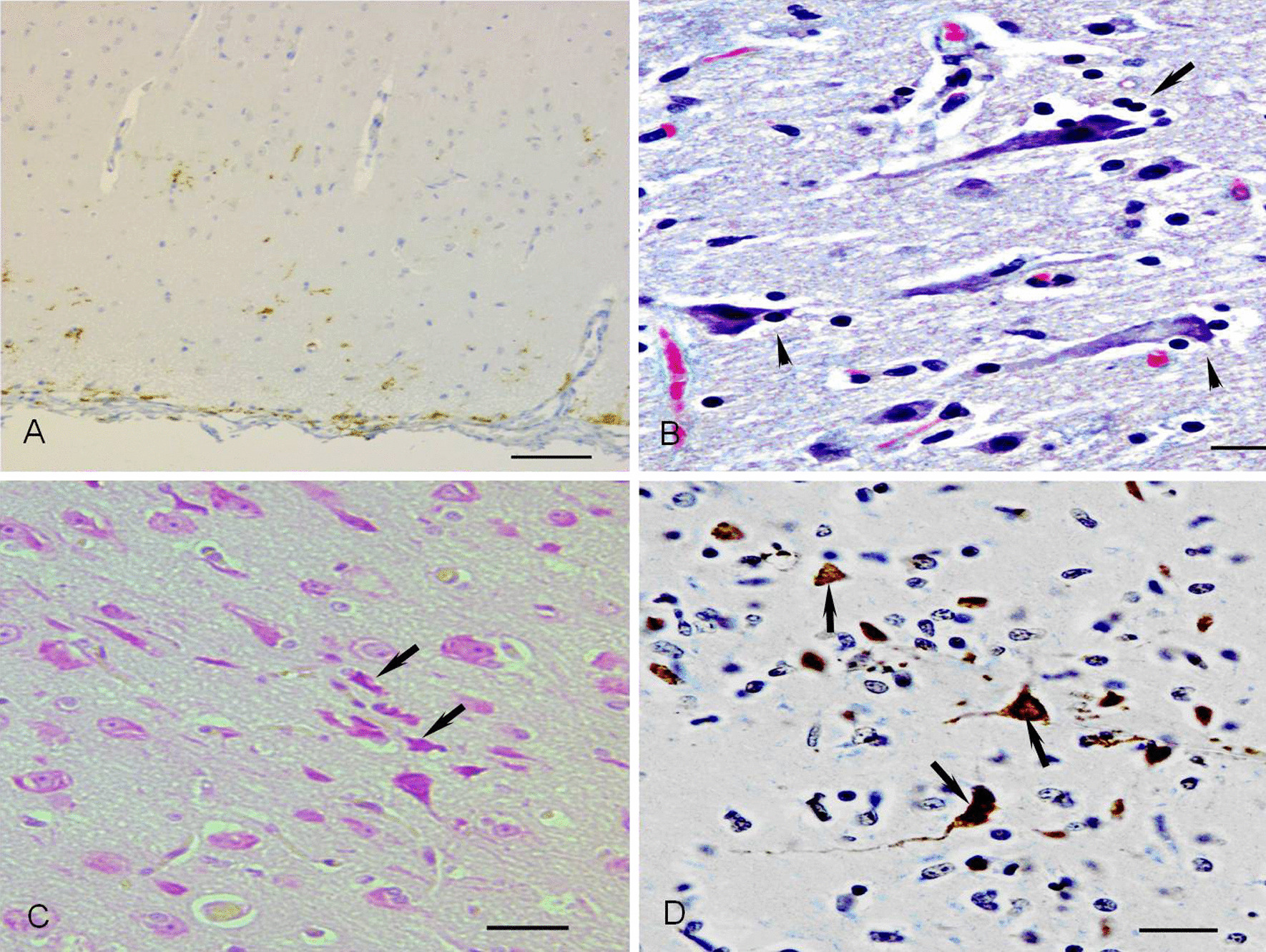
mild CDV-infected pyramidal cells. **A** Lots of CDV-antigen positive cells were observed in the cerebral cortical layer. sABC staining, Bar = 100 μm. **B** The lesions characteristics of satellite phenomenon (up arrow) and neuronophagia(up arrow) are detected in CDV-infected pyramidal cells. HE staining, Bar = 20 μm. **C** Small shrunken pyramidal cells (up arrow) show dark red, situating in the apoptotic states. MGP staining, Bar = 20 μm. **D** Some apoptotic pyramidal cells (up arrow) with pyknosis appear strong positive reaction staining dark brown. TUNEL staining, Bar = 20 μm

The light CDV-infected neurons were pyramidal cells and Purkinje cells. When the infection was serious, the whole cortical matter was infected by CDV, in which lots of CDV-infected positive cells (Fig. 4A), including cells situated in meninges, molecular, external granular, and pyramidal layers, were detected by anti-CDV staining. The pyramidal cells located in grey matter, especially the large ones, were often infected by CDV, in which CDV-infected pyramidal cells became to degeneration showing satellite phenomenon and neuronophagia (Fig. 4B), or were shrunken and small with condensed nuclei stained dark red (Fig. 4C) by MGP staining. Some of the pyramidal cells were positive reactions for anti-CDV immunostaining and exhibited apoptosis (Fig. 4D)stained by TUNEL staining, indicating that the pyramidal cells severely infected by CDV were prone to be apoptotic cells. Following apoptosis of some pyramidal cells, big vacuole-like demyelination was observed in brain white matter, particularly under brain grey matter. Furthermore, a few Purkinje cells were also infected by CDV, showing that Shrunken cells with condensed nuclei, in which the chromatin was compacted in the periphery of the nuclei, had a positive reaction for anti-CDV staining in the Purkinje cell layer.

## Discussion

### Relationship between apoptosis and demyelinating pathogenesis in brain tissues

Brain tissue demyelination in CDV-infected dogs is a common encephalopathy and can be considered a characteristic encephalic lesion caused by CDV. Some studies have investigated the underlying mechanism of demyelination in dogs with CD and found that nerve fiber demyelination is mainly related to astrocytes and oligodendrocytes. The present study proved that apoptosis of neurons and glial cells is related to demyelination in brain tissues. Several studies [13, 27, 28] demonstrate that astrocyte is the main target of CDV in the CNS. In the white matter, astrocytes surrounded by myelin sheath positively affect neuronal and oligodendroglial survival. Infected astrocytes fail to produce neurotrophic factors required for neurons and oligodendrocytes. Thus, CDV-infected astrocytes may have an important function in the mechanism of demyelination. Previous studies [29, 30] proved that astrocytes were the principal CDV-infected target in brain tissues and had a strong positive reaction for anti-CDV immunostaining. In this study, the metabolic disorder of astrocytes in CDV-infected dogs showed a strong positive reaction, but in control dogs, weak positive reactions stained by anti-GFAP immune technique were observed in the white matter, especially in demyelinating areas. Many astrocytes showed a strong positive reaction for TUNEL staining, indicating that the positive-reactive astrocytes had been in apoptosis. Like the measles virus [31], CDV can disrupt the glial fibrillary acidic protein cytoskeleton and cause astrocyte apoptosis. Thus, when several astrocytes died via apoptosis, neurotrophic factors were not transmitted to oligodendrocytes for myelin maintenance, and the demyelination process was prompted gradually.

Oligodendrocytes are directly related to demyelination of the brain in dogs with CD. In the demyelinating areas, negative regulation of myelin gene transcription, inhibition of specific enzymatic activity in oligodendrocytes [32], oligodendrocyte degeneration, and reduction of the population of oligodendrocytes suggest the involvement of oligodendrocytes in the acute distemper demyelination process [33]. This study found that the oligodendrocytes in the demyelinating area were disordered and lost normal structure situation. Multipolar mature oligodendrocytes decreased because of apoptosis. By contrast, bipolar oligodendrocyte precursor cells increased in the demyelinating area, showing a strong positive reaction for GalC with anti-GlaC staining. Pearce-Kelling et al. reported that bipolar oligodendrocyte exhibits high susceptibility for certain CDV strains [34].

Similarly, another bipolar cell population obtained from adult canine brains revealed an increased susceptibility for CDV in vitro studies [35]. A considerable number of CDV infections in white matter can result in oligodendroglial apoptosis that expressed strong positive cells by TUNEL staining in demyelinating parts. Oligodendrocytes can produce many substances that are necessary for the formation of the myelin sheath. Thus, they have a significant function in maintaining the integrity of myelin sheath. Furthermore, this study found apoptosis in many pyramidal cells in gray matter and neurons in nervous nuclei, indicating a strong positive reaction by TUNEL staining. Their axons were degenerated, dissolved, and died, which led to the disintegration of myelin sheaths around the axons and the formation of large vacuoles in the demyelinating areas [30].

### Relationship between apoptosis and CDV infection in brain tissues

Previous studies [29, 36–38] have proven that brain tissues are infected by CDV mainly through the bloodstream and cerebrospinal fluid. The present study demonstrated that the apoptotic cells in brain tissues were related to the CDV-infected route. The earlier the cell contact with CDV, the more serious the CDV infection was, the more obvious apoptosis was. According to this research, the blood-borne infection, in which many cells were infected, is earlier and more severe than cerebrospinal fluid infection. Krakowka et al. verified that neuron invasion of CDV occurs predominantly through the hematogenous route [39]. Summers et al. [40] and Axthelm et al. [41] demonstrated that viral antigen is first detected in the brain capillary tissues and venular endothelial at 5 and 6 days pi and/or perivascular lymphocytes, astrocytic foot processes, and pericytes at 8 days pi. The present research found that CDV followed by blood circulation could intrude the endothelium of small blood vessels in the brain tissues and replicated in there, resulting in lots of CDV in the endothelium, and a strong positive reaction was visualized for anti-CDV immunostaining. This pathological cellular lesion could finally lead to severe CDV-infected endothelium to death via apoptosis. The CDV in the endothelium of blood vessels could also infect the astrocytes via the astrocytic foot processes, resulting in serious infection of astrocytes and apoptosis, as demonstrated by anti-CDV and TUNEL immunostaining. With this pattern, CDV could destroy the blood–brain barrier, enter into the brain tissues, and infect other neurons and glial cells.

Vandevelde et al. investigated the infection route of CDV in the brain tissues and found that ependymal and subependymal white matter infections could be infected by CDV [42], suggesting that the brain tissues can be infected through the cerebrospinal fluid pathway. Moreover, a replicated-CDV infection of the choroid plexus endothelium with the release of progeny virus into the cerebrospinal fluid followed by ependymal infection and spread of the virus to the subependymal white matter can be observed at 10 days pi [42, 43]. The present study found that the pathological morphology of ependymal cells infected by CDV was changing from columnar to cubiform, then becoming flat cells, in which several eosinophilic inclusions showed a strong positive reaction in their cytoplasm by anti-CDV immunostaining. When the CDV infection was serious, many ependymal cells on the ventricular surface died via apoptosis and expressed intense positive by TUNEL staining. This phenomenon indicates that the CDV from the cerebrospinal fluid invaded the ependymal cells and replicated, resulting in ependymal cells to infection and apoptosis. In this approach, the CDV entered the subependymal white matter by destroying the ependymal membrane barrier. Thus, the demyelination around the ventricle was more serious than in the other parts of the white matter in brain tissues.

In addition, this research revealed that the neuronic apoptosis in nerve nucleus or grey matter and demyelination in white matter occurred randomly in brain tissues, and no particular district and position of brain tissue were as some constant region of the lesion through surveying the sections of the cerebrum, brain stem and cerebellum of dogs with CDV in different parts. So in clinical practice, no particular neurological manifestation could be used as a diagnostic symptom for dogs with CD, though many diverse nervous signs were examined clinically in CDV- infected dogs. On the side, it had been reported that neurological signs of dogs with CD were caused by other factors [13, 44].

## Conclusion

To our knowledge, this study is the first to show that natural CDV infection resulted in apoptosis in all kinds of brain tissue cells. The data obtained by HE, methyl green–pyronin, TUNEL technique, immune sABC, and immunohistochemistry supported that apoptosis had an important function in demyelinating pathogenesis. CDV could infect the endothelium of the brain vessel and the astrocytic food process that surrounded the blood vessels. A severe infection could lead to apoptosis, resulting in blood–brain barrier damage and viral entry into the grey and white matter. CDV could also invade the ependymal cells, induce apoptosis, damage the cerebroventricular barrier and intrude the white matter. CDV that entered the brain tissues could continue to infect the glial cells, a neuron in the nerve nucleus, pyramidal cells, and Purkinje cells, and cause some serious infecting cells to apoptosis. Thus, apoptosis in brain cells was mainly related to the route of transmission and cytotropism of CDV. The apoptosis of astrocytes, oligodendrocytes and some neurons play a significant role in the demyelinating pathogenesis in dogs with acute CD.

## Data Availability

All data generated or analyzed during this study are included in this published article.

## Abbreviations

CDV: Canine distemper virus
CD: Canine distemper
CNS: Central nervous system
HE: Hematoxylin-eosin
DAB: Diaminobenzidine
TUNEL: TdT mediated dUTP nick end labeling
MGP: Methyl green–pyronin
NP: Nucleocapsid protein
PBS: Phosphate buffer saline
RT: Room temperature

## References

[1] Pan YQ, Liu XY, Meng LP, Zhu GR, Xia YK (2013). Pathogenesis of demyelinating encephalopathy in dogs with spontaneous acute canine distemper. J Inte Agric.

[2] Avila M, Alves L, Khosravi M, Ader-Ebert N, Origgi F (2014). Molecular determinants defining the triggering range of prefusion f complexes of canine distemper virus. J Virol.

[3] Tshering D, Tenzin T, Kuenga T, Dawa T, Karma R (2020). Seroprevalence and risk factors of canine distemper virus in the pet and stray dogs in Haa, western Bhutan. BMC Vet Res.

[4] Duque-Valencia J, Sarute N, Olarte-Castillo XA, Ruíz-Sáenz J, Duque-Valencia J (2019). Evolution and interspecies transmission of canine distemper virus—an outlook of the diverse evolutionary landscapes of a multi-host virus. Viruses.

[5] Santiago RM, Renata FB, Cláudio WC, Julian RS (2019). Tropism and molecular pathogenesis of canine distemper virus. Virol J.

[6] Mariana MZM, Thalita ESO, Nayara EV, Wanderlei M, Zalmir SC (2020). Immunohistochemical evidence of canine morbillivirus (canine distemper) infection in coatis (Nasua nasua) from Southern Brazil. Transbound Emerg Dis.

[7] Roberto RCM, Fernando MH, Nidia AC, Osvaldo LD, Claudia IMG (2020). Canine distemper in neotropical procyonids: Molecular evidence, humoral immune response and epidemiology. Virus Res.

[8] Deem SL, Spelman LH, Yates RA, Montali RJ (2000). Canine distemper in terrestrial carnivores: a review. J Zoo Wildl Med.

[9] Mike D (2014). Risk of re-emergence of canine distemper. Vet Rec.

[10] Dong KY, Ha HK, Siu L, Yoon SY, Jungwon P (2020). Isolation and molecular characterizations of canine distemper virus from a naturally infected Korean dog using Vero cells expressing dog signaling lymphocyte activation molecule. For Vet Sci..

[11] Ruffalo G, Byers M, Ward MP (2020). A canine distemper virus seroprevalence study of dogs on three islands in the Torres Strait region, Australia. Aust Vet J.

[12] Alice B, Bianca Z, Alice F, Alessia S, Silvia O (2020). Two waves of canine distemper virus showing different spatio-temporal dynamics in Alpine wildlife (2006–2018). Infect Genet Evol.

[13] Tatianna FSN, Paulo HLB, Priscila AB, Danísio PM, Gisele FM (2020). Contribution of astrocytes and macrophage migration inhibitory factor to immune-mediated canine encephalitis caused by the distemper virus. Vet Immunol Immunopathol.

[14] Frauke S, Seham AA, Barbara BR, Verena H, Christina P (2016). Accumulation of extracellular matrix in advanced lesions of canine distemper demyelinating encephalitis. PLoS ONE.

[15] Carvalho OV, Botelho CV, Ferreira CG, Scherer PO, Soares-Martins JA (2012). Immunopathogenic and neurological mechanisms of canine distemper virus. Adv Virol.

[16] Beineke A, Puff C, Seehusen F, Baumgartner W (2009). Pathogenesis and immunopathology of systemic and nervous canine distemper. Vet Immunol Immunopathol.

[17] Martella V, Elia G, Buonavoglia C (2008). Canine distemper virus. Vet Clin North Am Small Anim Pract.

[18] Lauren G, Laurie C, Margaret M, Eric G (2020). Distemper encephalomyelitis presenting with lower motor neuron signs in a young dog. J Am Anim Hosp Assoc.

[19] Luane LP, Ana RL, Danielli MM, Edivaldo HO, Michel PS (2019). Mesenchymal stem cells in dogs with demyelinating leukoencephalitis as an experimental model of multiple sclerosis. Heliyon.

[20] Amude AM, Alfieri AA, Alfieri AF (2007). Clinicopathological findings in dogs with distemper encephalomyelitis presented without characteristic signs of the disease. Res Vet Sci.

[21] Moro L, Martins AS, Alves CM, Santos FGA, Del Puerto HL (2003). Apoptosis in the cerebellum of dogs with distemper. J Vet Med.

[22] Moro L, Martins AS, Alves CM, Santos FGA, Nunes JES (2003). Apoptosis in canine distemper. Arch Virol.

[23] Del Puerto HL, Martins AS, Moro L, Milsted A, Alves F (2010). Caspase-3/-8/-9, Bax and Bcl-2 expression in the cerebellum, lymph nodes and leukocytes of dogs naturally infected with canine distemper virus. Genet Mol Res.

[24] Guo A, Lu C (2000). Canine distemper virus causes apoptosis of Vero cells. J Vet Med.

[25] Del Puerto HL, Martins AS, Braz GF, Alves F, Heinemann MB (2011). Vero cells infected with the Lederle strain of canine distemper virus have increased Fas receptor signaling expression at 15 h post-infection. Gen Mol Res.

[26] Helen L, Del P, Almir SM, Amy M, Elaine MSF (2011). Canine distemper virus induces apoptosis in cervical tumor derived cell lines. J Virol.

[27] Mutinelli F, Vandevelde M, Griot C, Richard A (1988). Astrocytic infection in canine distemper virus-induced demyelination. Acta Neuropathol.

[28] Klemens J, Ciurkiewicz M, Chludzinski E, Iseringhausen M, Klotz D (2019). Neurotoxic potential of reactive astrocytes in canine distemper demyelinating leukoencephalitis. Sci Rep.

[29] Watanyoo P, Angeline PPT, Araya R, Nopadon P, Nguyen TL (2017). Expression of canine distemper virus receptor nectin-4 in the central nervous system of dogs. Sci Rep.

[30] Lucá R, Giacominelli-Stuffler R, Mazzariol S, Roperto S, Cocumelli C (2017). Neuronal and astrocytic involvement in striped dolphins (Stenella coeruleoalba) with morbilliviral encephalitis. Acta Virol.

[31] Duprex WP, Mcquaid S, Rima BK (2000). Measles virus-induced disruption of glial-fibrillary-acidic protein cytoskeleton in an astrocytoma cell line (U-251). J Virol.

[32] Glaus T, Griot C, Richard A, Althaus U, Herschkowitz N (1990). Ultrastructural and biochemical findings in brain cell cultures infected with canine distemper virus. Acta Neuropathol.

[33] Schobesberger M, Zurbriggen A, Summerfield A, Vandevelde M, Griot C (1999). Oligodendroglial degeneration in distemper: apoptosis or necrosis?. Acta Neuropathol.

[34] Pearce-Kelling S, Mitchell WJ, Summers BA, Appel MJ (1991). Virulent and attenuated canine distemper virus infectsmultiple dog brain cell types in vitro. Glia.

[35] Orlando E, Imbschweiler I, Gerhauser I, Baumga RW, Wewetzer K (2008). In vitro characterisation and preferential infection by canine distemper virus of glial precursors with Schwann cell characteristics from adult canine brains. Neuropathol Appl Neurobiol.

[36] Seehusen F, Baumgärtner W (2010). Axonal pathology and loss precede demyelination and accompany chronic lesions in a spontaneously occurring animal model of multiple sclerosis. Brain Path.

[37] Wyss-Fluehmann G, Zurbriggen A, Vandevelde M, Plattet P (2010). Canine distemper virus persistence in demyelinating encephalitis by swift intracellular cell-to-cell spread in astrocytes in controlled by the viral attachment protein. Acta Neuropathol.

[38] Ludlow M, Nguyen DT, Silin D, Lyubomska O, de Vries RD (2012). Recombinant canine distemper virus strain Snyder Hill expressing green or red fluorescent proteins causes meningoencephalitis in the ferret. J Virol.

[39] Krakowka S (1989). Canine distemper virus infectivity of various blood fractions for central nervous system vasculature. J Neuroimmunol.

[40] Summers BA, Appel MJ (1987). Demyelination in canine distemper encephalomyelitis: an ultrastructural analysis. J Neurocytol.

[41] Axthelm M, Krakowka S (1987). Canine distemper virus: the early blood-brain barrier lesion. Acta Neuropathol.

[42] Vandevelde M, Zurbriggen A, Higgins RJ, Palmer D (1985). Spread and distribution of viral antigen in nervous canine distemper. Acta Neuropathol.

[43] Higgins RJ, Krakowka SG, Metzler AE, Koestner A (1982). Experimental canine distemper encephalomyelitis in neonatal gnotobiotic dogs. A sequential ultrastructural study. Acta Neuropathol.

[44] Selim C, Selçuk Ö, Şükrü D (2020). Canine distemper virus induces downregulation of GABA A, GABA B, and GAT1 expression in brain tissue of dogs. Arch Virol.

